# Nanodelivery of antiretroviral drugs to nervous tissues

**DOI:** 10.3389/fphar.2022.1025160

**Published:** 2022-11-08

**Authors:** Sodiq Kolawole Lawal, Samuel Oluwaseun Olojede, Oluwaseun Samuel Faborode, Okikioluwa Stephen Aladeyelu, Matome Nadab Matshipi, Sheu Oluwadare Sulaiman, Edwin Coleridge Stephen Naidu, Carmen Olivia Rennie, Onyemaechi Okpara Azu

**Affiliations:** ^1^ Discipline of Clinical Anatomy, School of Laboratory Medicine and Medical Sciences, Nelson R. Mandela School of Medicine, University of KwaZulu-Natal, Durban, South Africa; ^2^ Department of Physiology, School of Laboratory Medicine and Medical Sciences, Westville Campus, University of KwaZulu-Natal, Durban, South Africa; ^3^ Department of Physiology, Faculty of Basic Medical Sciences, Bingham University, Karu, Nasarawa State, Nigeria; ^4^ Department of Physiology, School of Medicine and Pharmacy, College of Medicine and Health Sciences, University of Rwanda, Huye, Rwanda; ^5^ Graduate Program in Cell Biology, Institute of Biological Sciences, Federal University of Minas Gerais (UFMG), Belo Horizonte, Brazil; ^6^ Department of Human, Biological and Translational Medical Sciences, School of Medicine, Hage Geingob Campus, University of Namibia, Windhoek, Namibia

**Keywords:** human immunodeficiency virus, blood-brain barrier, neurological disorders, silver nanoparticles, metabolic disorder

## Abstract

Despite the development of effective combined antiretroviral therapy (cART), the neurocognitive impairments associated with human immunodeficiency virus (HIV) remain challenging. The presence of the blood-brain barrier (BBB) and blood-cerebrospinal fluid barrier (BCFB) impedes the adequate penetration of certain antiretroviral drugs into the brain. In addition, reports have shown that some antiretroviral drugs cause neurotoxicity resulting from their interaction with nervous tissues due to long-term systemic exposure. Therefore, the research into the effective therapeutic modality that would cater for the HIV-associated neurocognitive disorders (HAND) and ART toxicity is now receiving broad research attention. Thus, this review explores the latest information in managing HAND using a nanoparticle drug delivery system (NDDS). We discussed the neurotoxicity profile of various approved ART. Also, we explained the applications of silver nanoparticles (AgNPs) in medicine, their different synthesis methods and their interaction with nervous tissues. Lastly, while proposing AgNPs as useful nanoparticles in properly delivering ART to enhance effectiveness and minimize neurocognitive disorders, we hypothesize that the perceived toxicity of AgNPs could be minimized by taking appropriate precautions. One such precaution is using appropriate reducing and stabilizing agents such as trisodium citrate to reduce silver ion Ag + to ground state Ag^0^ during the synthesis. Also, the usage of medium-sized, spherical-shaped AgNPs is encouraged in AgNPs-based drug delivery to the brain due to their ability to deliver therapeutic agents across BBB. In addition, characterization and functionalization of the synthesized AgNPs are required during the drug delivery approach. Putting all these factors in place would minimize toxicity and enhance the usage of AgNPs in delivering therapeutic agents across the BBB to the targeted brain tissue and could cater for the HIV-associated neurocognitive disorders and neurotoxic effects of antiretroviral drugs (ARDs).

## 1 Introduction

The concept of nanotechnology was first introduced in 1959 by an American physicist called Richard Feynman and is one of the most promising technologies in recent times (Bayda et al., 2019). The term “nanotechnology,” first used and defined by a Japanese scientist, Norio Taniguchi, consists of the separation, consolidation or breaking down of materials by one atom or molecule (Taniguchi, 1974).

The advancement in technology has brought about nanotechnology, the application of technology to harness the use of nanomaterials. Nanotechnology is an appealing field with broad potential and wide applications, having contributed to the fabrication of more potent and sophisticated materials, wastewater recycling, aspects of drug development, diagnosis and treatment of diseases, and advanced information and communication tools (Benelmekki, 2015; Cheon et al., 2019). Nanotechnology deals with the manufacturing and manipulation of nano-sized materials known as nanoparticles. A branch of nanotechnology that involves diagnosing, treating, and preventing diseases with nanoparticles and molecular devices to enhance human well-being is known as nanomedicine (Hosseini et al., 2016; Vahedifard and Chakravarthy, 2021). Nanomedicine entails using nanoparticles to diagnose and ensure targeted delivery and controlled release of therapeutic agents and active materials in living cells (Patra et al., 2018).

Nanoparticles (NPs) are nano-size particles that range between 1 and 100 nm in size. NPs exhibit unique properties due to their dimensions, making them suitable candidates for fabricating other complex nanoconjugates (Benelmekki, 2015). Thus, they recently received broad research attention and biomedical application. NPs are mostly complex particulates with three layers arranged from the innermost to the outermost (Khan et al., 2019), while some are single-layer particulate (Chundi et al., 2020). The innermost core layer represents the central part of the NPs. The middle shell layer is chemically different from other layers, and the outmost surface layer undergoes functionalisation with various macromolecules (Shin et al., 2016).

### 1.1 Types of nanoparticles

**FIGURE 1:**
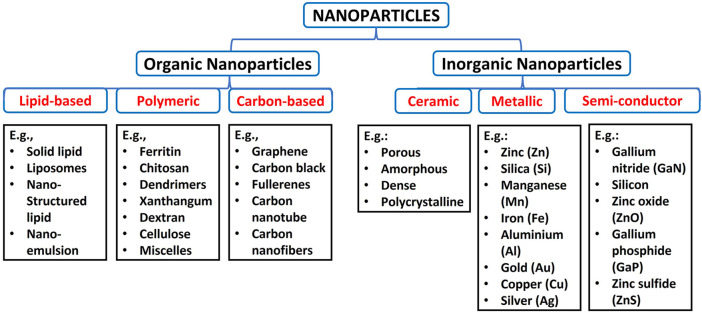
Types of nanoparticles, subdivisions, and examples of each subdivision.

Nanoparticles are classified into various classes based on their physicochemical property, morphology, and size. On this account, there are organic and inorganic nanoparticles. The organic nanoparticles are lipid-based, polymeric and carbon-based, while inorganic nanoparticles are grouped into ceramic, metallic and semiconductor nanoparticles, as shown in Figure 1 (Patra et al., 2018; Khan et al., 2019).

The most common polymeric NPs are ferritin, chitosan, dendrimers, xanthan gum, dextran, liposomes, cellulose, micelles, and alginate (Patra et al., 2018). Carbon-based are well known for excessive strength, versatility, electron affinity and good electrical conductivity and have received significant attention in biomedical applications (Astefanei et al., 2015; Maiti et al., 2018). Carbon-based NPs include graphene, carbon black, fullerenes, carbon nanotube, and carbon nanofibers (Anu Mary Ealia & Saravanakumar, 2017). The lipid-based NPs have lipid moieties as a solid core, a matrix filled with lipophilic molecules and an outer core stabilized by emulsifiers and surfactants (Rawat et al., 2011; Khan et al., 2019). Examples of lipid-based NPs are solid lipid nanoparticles, liposomes, nanostructured lipid carriers and nano-emulsions (Garcia-Pinel et al., 2019).

Ceramic-based materials are inorganic-based NPs with high thermal resistance and chemical inactivity (Khan et al., 2019). These NPs exhibit various forms like porous, amorphous, dense and polycrystalline. They are usually produced by heating carbides, phosphates, oxides and carbonates, followed by cooling (Bhardwaj et al., 2021). Semiconductor NPs represent other inorganic nanoparticles with smaller sizes, high resistivity, luminescence, quantum size effects, less weight, high surface area, and nonlinear optical activity (Tiwari and Rohiwal, 2019; Ishtiaq et al., 2020). These NPs are found in the periodic table groups II, III, IV, V, and VI. They include Gallium nitride (GaN), silicon, Cadmium telluride (CdTe), Indium phosphide (InP), zinc oxide (ZnO), indium arsenide (InAs), germanium, Cadmium sulfide (CdS), Gallium phosphide (GaP), zinc sulfide (ZnS) and cadmium selenide (CdSe) (Goodilin et al., 2019). Metallic nanoparticles are derived from metals, alkali metals, alkali earth metals and noble metals. These NPs possess great optical and excellent electrical properties, high surface area to volume ratio, small size and localised surface plasmon resonance (LSPR) (Khan et al., 2019). Examples of metallic nanoparticles are aluminosilicates, zinc (Zn), cerium (Ce), silica (Si), titanium dioxide (Ti), manganese (Mn), nickel (Ni), iron (Fe), aluminium (Al), gold (Au), copper (Cu) quantum dots and silver (Ag) (Loomba & Scarabelli, 2013; Khan et al., 2019).

### 1.2 Applications of nanoparticles

Nanoparticles are swiftly transforming the field of nanotechnology with various applications owing to their special sizes and many other properties such as optical, chemical, mechanical and large surface area (Bhattacharyya et al., 2011; Khan et al., 2019). Among the variety of applications of nanoparticles are in wastewater treatment, prevention of environmental pollution, material remediation, mechanical and electronics, manufacturing, food industry, and medicine.

Nanoparticles that have attracted interest in wastewater treatment are carbon nanotubes, nanocomposites, zero-valent metal nanoparticles, and metal oxide nanoparticles (Oghyanous, 2021). This is due to their unique size and large surface area with catalytic, reactivity and adsorption activities (Schodek et al., 2009; Oghyanous, 2021). Similarly, NPs prevent environmental pollution due to their tunable shape and small size, which are suitable for sensing, preventing, and degrading environmental pollutants (Mehndiratta et al., 2013). Recently, nanocarriers have been used to deliver food additives to products without morphological alterations (Singh et al., 2017). In addition, nanoparticles were utilised as nanosensors to detect the presence of microbes and contaminants in food products (Bratovčić et al., 2015). Organic, inorganic and metallic nanoparticles have wide applications in drug delivery, management and prevention of diseases (Hassan et al., 2017; Rezaei et al., 2019). However, much attention has been directed to FDA-approved nanoparticles such as dendrimers, nanocrystals, liposomes, micelles, proteins, and polymers (Ventola, 2017).

### 1.3 Nanoparticles and blood-brain barrier

**FIGURE 2:**
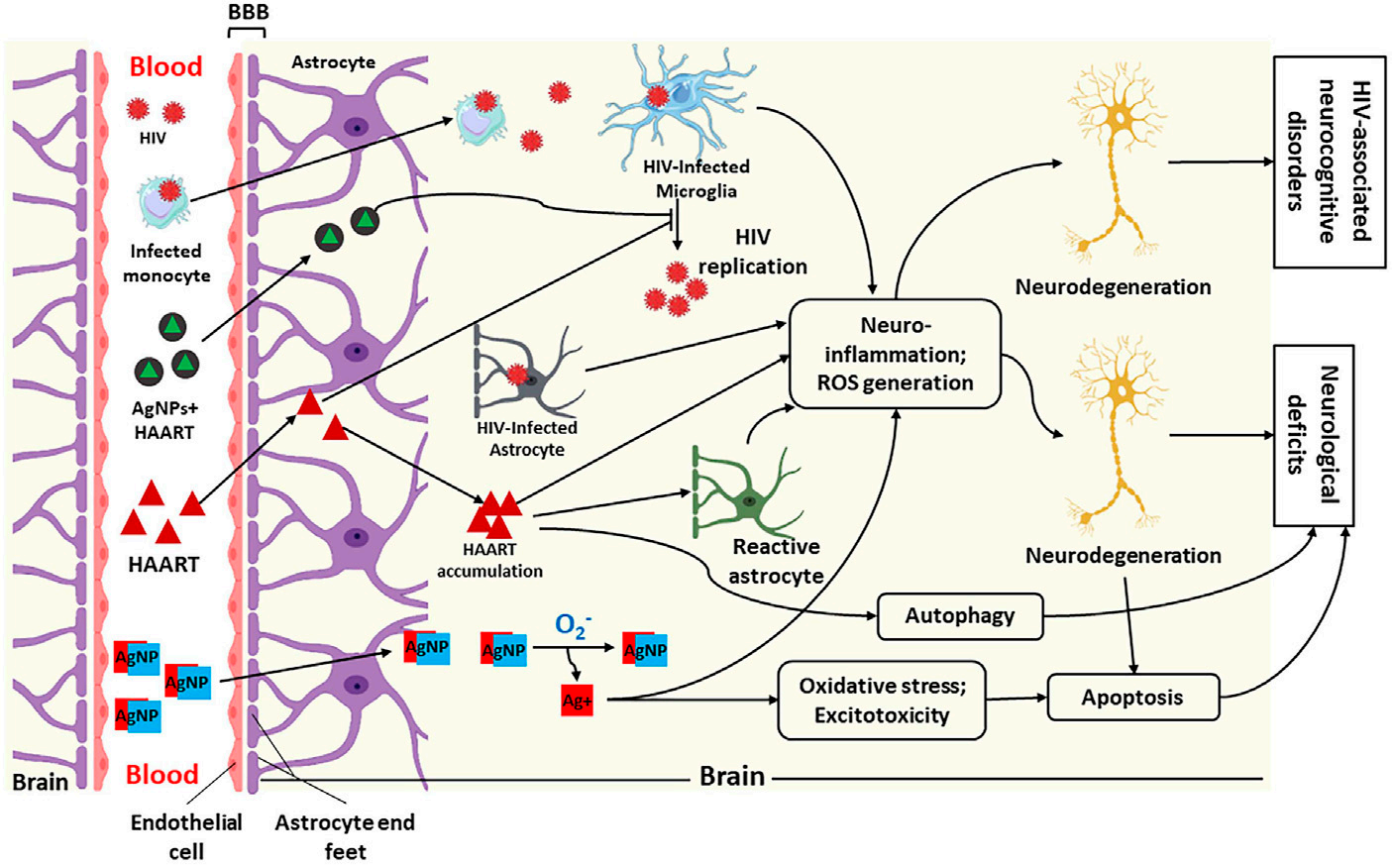
Illustration of penetration of silver nanoparticles (AgNPs), HIV and Highly Active Antiretroviral Therapy (HAART) + AgNPs (AgNPs + HAART) through the blood-brain barrier (BBB) and their possible mechanism of action within the brain. This figure shows that HIV and infected macrophages traverse the BBB into the brain interstitial space to infect microglia, leading to HIV replication. Furthermore, HAART and AgNPs + HAART traverse the BBB to block or inhibit HIV replication. Lastly, AgNPs alone can also cross BBB into the brain. Within the brain, AgNPs get oxidised to release Ag^+^. The infected microglia, astrocyte, and Ag^+^ cause neuroinflammation and increased reactive oxygen species (ROS) generation, leading to neurodegeneration and HIV-associated neurocognitive disorders. While HAART inhibits HIV replication, it also causes neurodegeneration *via* neuroinflammation, ROS generation and autophagy. On the contrary, AgNPs + HAART show a better output by preventing HIV replication with reduced neurocognitive disorders. Adapted from (Lawal et al., 2022a; Lawal et al., 2021; Lawal et al., 2022b).

The treatment of various neurological disorders has been difficult due to the blood-brain barrier (BBB) and different side effects (Warren, 2018). The BBB is a barrier between cerebral capillary blood and the brain’s interstitial fluid. It composes of basement membrane and endothelial cells, neuroglial membrane, and the projections of astrocytes. These three (3) components impede the entry of various substances into the central nervous system (Dotiwala et al., 2020). Most viruses, including HIV, can navigate BBB shortly after the primary infection (E. Rahimy et al., 2017). However, BBB impedes antiretroviral drug penetration, resulting in a poor outcome in treating HIV-associated neurocognitive disorders (HAND), as illustrated in Figure 2 (Osborne et al., 2020). The ability of nanoparticles to cross the BBB has been identified as a therapeutic approach to reducing CNS diseases (Cena and Jativa, 2018). Similarly, antiretroviral drugs (ARDs) coupled with nanoparticles have been identified as a solution for reducing HIV in the CNS due to their ability to improve the ARDs tissue distribution and bioavailability, leading to reduced adverse and toxic effects of HIV (Bowen et al., 2020).

### 1.4 General methods for nanoparticle synthesis

**FIGURE 3:**
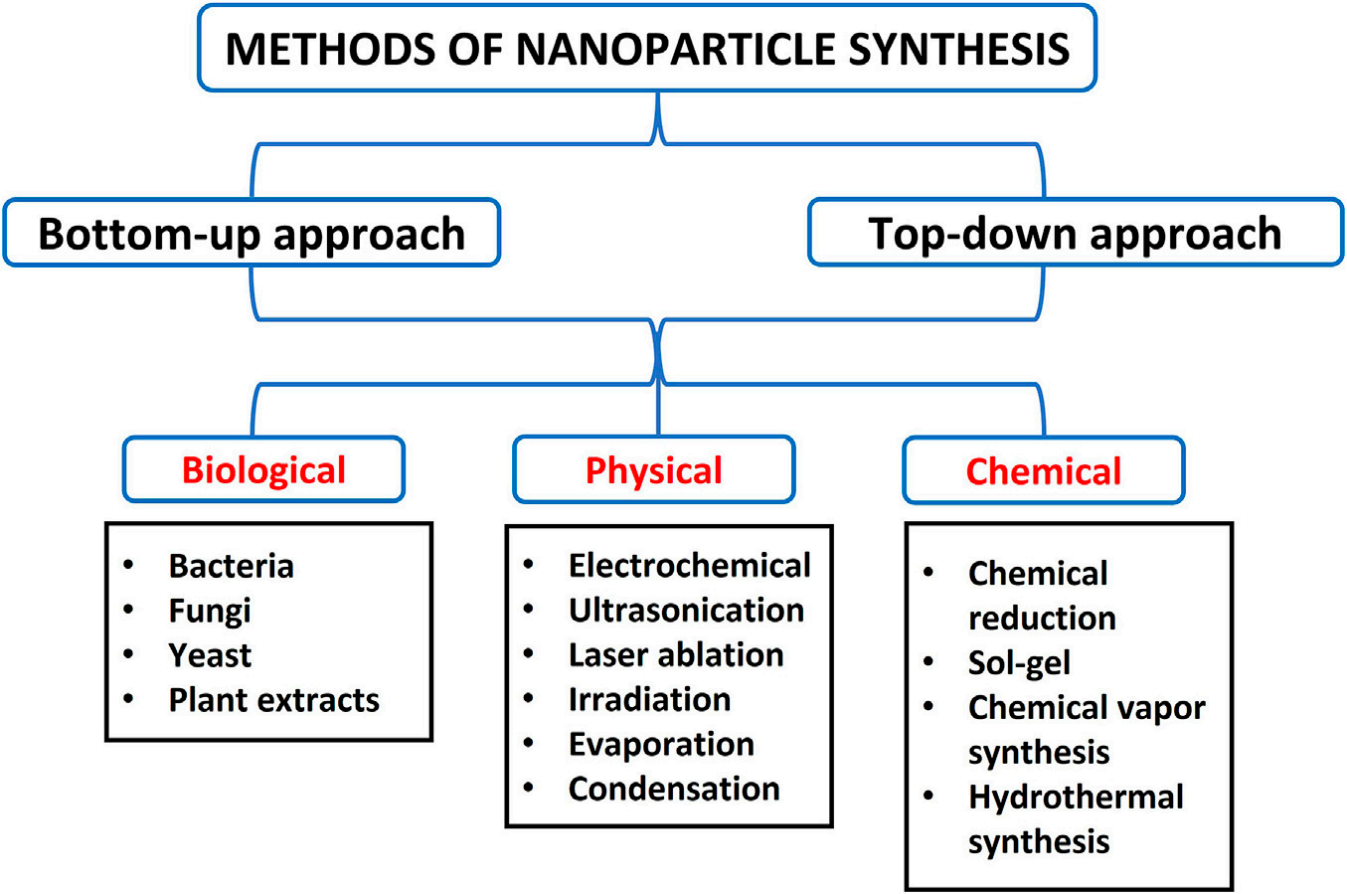
Summary of different methods of synthesising nanoparticles.

Various methods have been developed to synthesise NPs, but these approaches are majorly grouped into two (2): bottom-up and top-down processes. The bottom-up method involves building up atomic constituents, while the top-down approach requires breaking bulk materials into minute components (Rajput, 2015). These approaches are further divided into biological, chemical, and physical methods with subdivisions such as gas condensation, electrodeposition, sol-gel technique, vacuum deposition/vapourisation, mechanical attrition, chemical precipitation, and chemical vapour condensation (Rajput, 2015; Sakhare et al., 2022). Of these techniques, metallic and inorganic NPs are usually prepared by chemical methods, gas condensation, and the sol-gel process. Also, semiconductor NPs and nanocrystalline are typically synthesised using chemical vapour condensation, while ceramics and alloys are manufactured using mechanical attrition (Rajput, 2015). Furthermore, the synthesis of metallic nanoparticles is broadly classified into top-down approaches by utilising physical, biological or chemical methods and bottom-up approaches, as shown in Figure 3 (Thakkar et al., 2010).

#### 1.4.1 Synthesis of silver nanoparticles

Various approaches to synthesising AgNPs, such as physical, chemical, microemulsion, sonoelectrochemistry, photoinduced reduction, electrochemical synthetic, irradiation method, UV-initiated photoreduction, and microwave-assisted techniques were described (Iravani et al., 2014). The physical method of preparing AgNPs utilises physical energies with resultant AgNPs of narrow size dimensions. Also, this technique synthesises a large amount of AgNPs during a single run (Singh and Kaur, 2019). The physical approach involves lithography, laser ablation, gamma irradiation, evaporation-condensation and electrical irradiation, which is broadly employed in preparing AgNPs (Güzel and Erdal, 2018).

Biosynthesis of AgNPs is another method that has attracted significant research interest and wide application because they are eco-friendly, easy to produce, and cost-effective. Biosynthesis (green synthesis) involves using medicinal plant extracts, herbs, and microorganisms like fungi, bacteria, yeast and algae to prepare AgNPs (Güzel and Erdal, 2018). The ready availability of medicinal plants and various essential phytochemicals and functional constituents that can reduce the silver ions was described as another reason the green synthesis is receiving broad attention. In addition, most of these medicinal plants contain active compounds such as amino acids, saponins, polysaccharides, flavones, proteins, tannins, enzymes, terpenoids and vitamins (Güzel and Erdal, 2018).

Chemical reduction is the most widely applied in preparing silver nanoparticles. In this technique, the precursor (silver nitrate, AgNO_3_) is reduced to Ag^0^ by using reducing agents like sodium citrate, hydrogen, tollens, trisodium citrate, ascorbate, ethylene glycol-block copolymers, sodium borohydride (NaBH_4_), and N, N-dimethylformamide (DMF) (Iravani et al., 2014). Also, materials like sodium carboxyl methylcellulose (NaCMC), starch, polyvinyl pyrrolidone (PVP K 30), polysaccharides, bovine serum albumin, peptide, starch, and chitosan have been employed as the stabilising or capping agents in preparing AgNPs (Zewde et al., 2016; Patel et al., 2017). The production of metallic silver precedes the agglomeration of metallic silver into oligomeric clusters, and subsequently, the metallic silver nanoparticle is formed (Iravani et al., 2014). The reducing and stabilising agents play an essential function in the stability of the manufactured AgNPs and the shape and size of AgNPs (Iravani et al., 2014). It is important to note that chemical methods of preparing AgNPs have synthesised different shapes ranging from cylinders, wires, cubic, rods, triangular, bars, pyramidal, prisms and spherical (Gamboa et al., 2019).

Recent studies have utilised silver nitrate (AgNO3) and trisodium citrate to successfully synthesise the AgNPs (Lawal et al., 2021). Briefly, an aqueous solution of silver nitrate (AgNO_3_) was prepared from AgNO_3_ crystal. Then, a desired stock aqueous solution of trisodium citrate (TSC) was prepared from crystal trisodium citrate and used as a reducing and stabilising agent. Then, the synthesized AgNPs were characterized with various concentrations (from 0.5 to 2 M) of TSC and mixed with AgNO_3._ The solution was stirred continuously for 5 min at 90°C. The resultant solution of the mixtures was adjusted with concentrated NaOH at a pH of 10.5. A colour change from colourless to amber-yellow was used to indicate a successful AgNPs synthesis.

#### 1.4.2 Summary of silver nanoparticles synthesis-a new method

The first step is to prepare AgNPs using the chemical method, where the precursor will be silver nitrate and trisodium citrate as stabilising, reducing and capping agents. A small quantity of silver nitrate as low as 0.03 mol/dm^3^ will be mixed with 0.1 mol/dm^3^ of trisodium citrate and heated for 90 min.

The mixture will be centrifuged at 12,000–15,000 rpm for about 15–30 min, and the supernatant will be discarded. The mixture will then be redispersed in an aqueous solution and stored at pH 10.5 in NaOH. The colour change to an amber colour will indicate the formation of AgNPs.

After successfully synthesising AgNPs, the characterisation will follow using HR-TEM and HR-SEM for shape and size. Absorption peak by UV-vis spectroscopy. Energy dispersive X-ray analysis to examine the elemental composition. Raman Spectroscopy to show chemical structure, crystallinity and molecular interactions and zeta potential to show the surface charges.

Shortly after that, the appropriate amount of the antiretroviral drugs (powdered form) will be dissolved in the appropriate medium (water or alcohol, depending on the nature of the drugs) and mixed with the synthesised AgNPs. The mixture will be continuously stirred in ultra-sonication and centrifuge at 4,500 rpm for about 40–50 min at 40°C.

The nanoconjugate will then be evaluated for proper conjugation by UV-Vis spectroscopy. Fourier transform infrared spectroscopy will be used to examine the absorption and functional groups, while the elemental composition of the nanoconjugates will be determined by Energy dispersive X-ray. Also, Raman Spectroscopy to show chemical structure, crystallinity and molecular interactions (Lawal et al., 2021). More importantly, any retroviral drugs can be used with this new approach. This is based on the fact that AgNPs exhibit a large surface area to volume ratio, one of the essential features in the drug delivery system. For Example, Tenofovir disoproxil fumarate was coupled successfully with silver nanoparticles, and their effects were investigated in animal model experiments (Lawal et al., 2022a; Olojede et al., 2022). However, before considering loading any retroviral drugs using this new proposed approach, characterisation of the AgNPs to examine the morphology, shape, size, and elemental composition are all essential.

### 1.5 Silver nanoparticles

Silver is a fundamental and non-toxic element that forms the precursor of silver nanoparticles (AgNPs) with unique electrical and thermal properties (Chen and Schluesener, 2008). AgNPs are nanoparticles of sizes ranging from 1 to 100 nm on the nanoscale (Klaus et al., 1999). Silver nanoparticles represent one of the essential metallic nanoparticles with appealing characteristics that fuelled their wide application in biomedical sciences. AgNPs have been extensively used to diagnose, treat, and prevent diseases in nanomedicine. For example, AgNPs have received broad attention and great success in cancer detection and management (Zink et al., 2016).

#### 1.5.1 Characteristics of silver nanoparticles

Silver nanoparticles exhibit excellent physicochemical properties such as effective electrical conductivity, unique optical properties, potent thermal properties, and promising biological activities (Zhang et al., 2016a). These outstanding characteristics of AgNPs were ascribed to their large surface area to volume ratio, small size, and tunable shapes (Li et al., 2001; Sharma et al., 2009; Calderón-Jiménez et al., 2017; Ivanova et al., 2018). In addition, one of the optical properties of AgNPs is surface plasmon resonance (SPR) which entails the ability of AgNPs to receive and scatter light effectively. This process (SPR) occurs when electrons on the metal surface are excited and go through oscillation to absorb and disperse (Kim et al., 2007; Bansal et al., 2014; Keat et al., 2015). Interestingly, this property has been more significant in AgNPs than in other metals like copper and gold (Xia and Halas, 2005).

#### 1.5.2 Toxicity profile of silver nanoparticles

Silver nanoparticles are metallic nanoparticles ranging between 1 and 100 nm in size, with higher loading efficacy (Mahajan et al., 2012). Despite the benefits of silver nanoparticles, their toxicity is also well-documented (Lekamge et al., 2018; Ferdous and Nemmar, 2020). Studies have attributed AgNPs toxicity to one or both releases of silver ions in the cell or the method used in the AgNPs synthesis (Akter et al., 2018; Smith et al., 2018). The physiochemical properties of AgNPs (e.g., size, shape, concentration, agglomeration, or interaction with the biological system) are major contributors to their toxicity. These properties have been associated with mitochondrial dysfunction and oxidative injury (Akter et al., 2018). AgNPs toxicity mechanism is linked with the generation of excessive reactive oxygen species (ROS), cellular mitochondrial dysfunction, inflammatory response and cell apoptosis (Yu et al., 2020). Recently, a study reported that cardiac cells treated with AgNPs generate ROS and mitochondrial dysfunction, and mitochondrial ATP was reduced as implicated in metabolic disorders (Khan et al., 2021). Another study observed that neural cells can uptake AgNPs of 3–5 nm in size, as indicated by the expression of inflammatory genes and neurodegenerative disorders in murine brain astrocytes and microglia (Huang et al., 2015). AgNPs cross the BBB irrespective of the size and concentration to influence the brain cells, as observed after 28 days of 5 mg kg^−1^ BW AgNPs (20 and 200 nm) injection in the experimental Wistar rats (Tang et al., 2010). Interestingly, literature has reported that not all silvers are toxic. AgNPs ranging from 12 to 20 nm were not toxic in rats’ tissue. However, as the dose and concentration increased, harmful effects were observed (Sung et al., 2011; Smith et al., 2018).

#### 1.5.3 Application of silver nanoparticles in drug delivery

**FIGURE 4:**
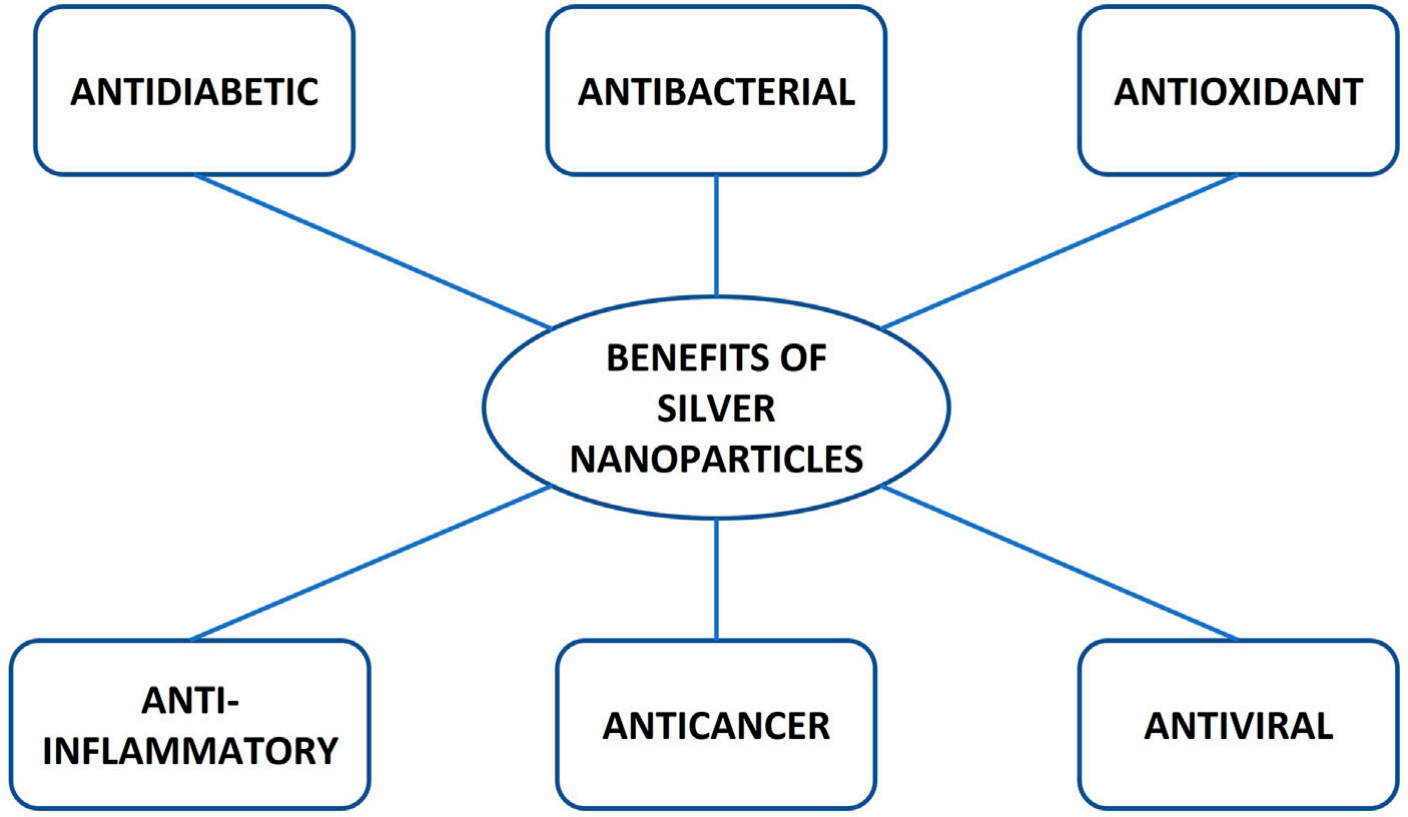
Benefits of silver nanoparticles.

AgNPs have received significant attention in diagnostics, drug delivery, the pharmaceutical industry, the food industry, cosmetics, nutraceuticals, and the manufacturing of biosensors (Chernousova and Epple, 2013). AgNPs are utilised in biomedical research to deliver therapeutic agents such as antiviral, antibacterial, antifungal, antidiabetic, anti-inflammatory, and antioxidant agents, as illustrated in Figure 4 (Alkaladi et al., 2014; Bedlovicova et al., 2020; Kwon and Koh, 2020). Of all the applications of silver nanoparticles, the antiviral, antidiabetic, anti-inflammatory and antioxidant properties are keys to alleviating HIV-associated neurocognitive disorders (HAND) and ART toxicity.

##### 1.5.3.1 Silver nanoparticles as an antiviral agent

Conventional antiviral drugs have been used to manage viral infections, but some agents may lead to physiological toxicity due to systemic exposure (Jeevanandam et al., 2022). Recently, silver nanoparticles have demonstrated an inhibitory property against pathogenic microbes, including viruses and bacteria (Jeevanandam et al., 2022). Several biomedical research studies have reported silver nanoparticles’ ability to effectively manage the viral infection and antiviral resistance (Das et al., 2020; Ratan et al., 2021). Silver nanoparticles exhibit unique properties such as the large surface area to volume ratio, surface plasmon resonance, size and shape-controlled production, which give them an advantage for coupling therapeutic agents to their surface (Khan et al., 2019; Loiseau et al., 2019).

A study has reported that the antimicrobial activities of silver nanoparticles are due to the presence of positive Ag ions (Ag+) that inhibit the growth of microorganisms *via* interaction with DNA, thereby causing denaturation of DNA and interruption of cell division (Kumar et al., 2016). Silver nanoparticles are used as an antiviral agent against HIV-1, monkeypox virus, Hepatitis virus, influenza, herpes simplex virus and respiratory syncytial virus (Khandelwal et al., 2014).

Several studies have experimented with using AgNPs as an antiviral agent and their cell toxicity profile. An *in vitro* experiment showed that the non-cytotoxic concentration of AgNPs exerted antiviral activities against HIV-1 (Sun et al., 2005). Another study reported that AgNPs interact with HIV-1 by biding to glycoprotein 120 knobs in a size-dependent manner (Elechiguerra et al., 2005). The antiviral properties of AgNPs are reported to be size-dependent. Previous studies have shown that smaller AgNPs of sizes between 5–20 nm can inhibit the replication of HIV (Khandelwal et al., 2014; Suganya et al., 2015; Ratan et al., 2021). Furthermore, small-medium AgNPs sized between 1 and 10 nm act on HIV-1 *via* binding to the disulfide bond regions of the CD4 domain located on the gp120 glycoprotein of the viral envelope (Elechiguerra et al., 2005). This shows that the size of silver nanoparticles influences the potency of their antiviral property.

A recent study has reported the potential benefits of using AgNPs as a treatment against SARS-CoV-2 because the metallic aspect of the nanoparticles interferes with the structural protein of SARS-CoV-2, thereby inhibiting it from binding to genetic material and preventing its replication (Das et al., 2020; Almanza-Reyes et al., 2021).

##### 1.5.3.2 Silver nanoparticles as an antidiabetic agent

AgNPs have been reported to demonstrate robust antidiabetic activity *via* effective inhibition against carbohydrate digestive and key enzymes of diabetes, including α-amylase and α-glucosidase (Balan et al., 2016). Furthermore, a study has reported that the AgNPs significantly inhibited non-enzymatic glycosylation compared with the aqueous leaf extract of *Pouteria sapota* (Prabhu et al., 2018). A similar study revealed that AgNPs possess antidiabetic activity by showing maximum inhibition of α-amylase at 78.84% and α-glucosidase at 58.86% at 100 μg/ml concentration (R and Scleeva P 2021). Several studies have reported that small-medium biosynthesised AgNPs control glycaemic index by significantly reducing the blood glucose levels in experimental rats (Agarwal et al., 2018; Alkaladi et al., 2014; S. Lawal et al., 2022b; Lawal et al., 2021). The mechanism of action of AgNPs on glycaemic control is linked with an increase in insulin secretion, which enhances hepatic glycogenesis and a significant increase in the expression of GLUT-2 levels in hepatic tissues (Alkaladi et al., 2014). Green synthesis of AgNPs with natural products such as medicinal plants has been evaluated for the potential antidiabetic activities and synergistic effects. Also, synthesised herbal-mediated AgNPs are the therapeutic target in treating type 2 diabetes due to their ability to inhibit protein tyrosine phosphatase 1B (PTP1B), which is the major negative regulator of the insulin signalling pathway (Shanker et al., 2017). Another study revealed *Pterocarpus marsupium* loaded with AgNPs was more effective for antidiabetic activity than *Pterocarpus marsupium* only (Bagyalakshmi and Haritha, 2017).

##### 1.5.3.3 Silver nanoparticles as an anti-inflammatory agent

Recent studies have demonstrated that AgNPs alone or in combination with therapeutic agents have excellent anti-inflammatory activity (Fehaid et al., 2020; Tyavambiza et al., 2021). AgNPs possess unique properties, such as a large surface area to volume ratio, which offer them an advantage of blocking the inflammation-enhancers like cytokines and inflammation-assisting enzymes (Agarwal et al., 2019). Green synthesis of AgNPs from leaf extracts has demonstrated an anti-inflammatory action in both *in vitro* and *in vivo* experiments (Agarwal et al., 2019). The mechanism of action of AgNPs green synthesis is attributed to their ability to reduce inflammatory cytokines such as tumour necrosis factor (TNF-α), interleukin 6 (IL-6) and interleukin 1β (IL-1β) (David et al., 2014). Furthermore, antiretroviral drugs coupled with AgNPs have been reported to offer an advantage in reducing the inflammatory effects of the drugs *via* reducing the inflammatory cytokines produced during the metabolism of the drugs (S. Lawal et al., 2022a; Olojede et al., 2021).

##### 1.5.3.4 Silver nanoparticles as an antioxidant agent

Antioxidants play a major role in controlling oxidative reactions by balancing the free radicals which attack macromolecules (such as lipids, proteins and nucleic acids), thereby preventing cell damage (Bratovcic, 2020). The accumulation of reactive species in the body is linked with diverse chronic pathologies, such as diabetes mellitus and neurological disorders (Volpe et al., 2018). Literature has reported the antioxidant activity of AgNPs *in vivo* and *in vitro* experiments (Jadczak et al., 2020; Keshari et al., 2020). A study reported a significant increase in antioxidant activities by AgNPs compared with Vitamin C (Keshari et al., 2020). The mechanism by which AgNPs act as antioxidant agents is not fully understood. However, studies have demonstrated that AgNPs activate the synthesis of intracellular enzymes such as superoxide dismutase (SOD), superoxide reductases (SOR), catalase (CAT) and reduced glutathione (GSH) which prevent oxidative damage in the body cell and tissues (Bedlovicova et al., 2020; Lawal et al., 2021). Moreover, AgNPs inhibit the reactive oxygen species by scavenging the free radicals *via* a decrease in hydroxyl and superoxide radicals (Guntur et al., 2018; Bedlovicova et al., 2020).

#### 1.5.4 Concentration and size of silver nanoparticles: The crucial factors in silver nanoparticles delivery system and activity

A great feature of nanoparticles is their large surface area which can be affected by several factors, including their size (1–100 nm) and aggregation, thereby affecting their activity. Studies have shown that the effect/activity of AgNPs depends on their size. For example, a study showed that AgNPs of the smallest size (10 nm) were more toxic to cells than larger sizes of 20–80 nm (Ivask et al., 2014). AgNPs attenuated the morphological switch and biofilm formation of some opportunistic pathogenic yeasts in a size-dependent manner. However, the smallest (7 nm)-sized AgNPs caused the highest attenuation rate (Szerencsés et al., 2020). In terms of the nanoparticle delivery system, the particle size is the essential factor that influences AgNPs delivery into the body because the size affects AgNPs cellular uptake, transportation and accumulation (Wu et al., 2019). In this regard, Wu et al. demonstrated that AgNP entry into B16 cells is size and time-dependent. Five-nanometer (5 nm) AgNPs were detected at 30 min, while 20 and 50 nm were detected in the cytoplasm and nucleus after 2 h. On the contrary, 100 nm AgNPs were only observed in the nucleus 12 h after incubating them with B16 *in vitro* (Wu et al., 2019).

Furthermore, Wu et al. (2019) experiments also showed that while AgNPs of lesser size entered the cell cytoplasm and nucleus earlier than the larger sizes, the uptake efficiencies (partly measured by the rate of entry and exit of the AgNPs through the cell) were 40.3 ± 7.6%, 22.0 ± 1.5%, 52.3 ± 4.7%, and 76.2 ± 8.0% for 5, 20, 50, and 100 nm AgNPs, respectively at 12 h. At 24 h, the efficiency was 58.5% ± 8.5%, 34.2% ± 8.3%, 57.9% ± 2.5% and 66.1% ± 9.7% for 5, 20, 50 and 100 nm AgNPs, respectively. At 5 nm, the AgNPs were more toxic to the cells, while at 100 nm, the AgNPs might not be able to enter the cellular nucleus and cause the expected regulation of cellular functions.

In another research, Bélteky et al. (2021) concluded that although smaller particles might have greater biological activity, they might become ineffective soon as they exit the cell. In comparison, larger particles might have a long-lasting effect. Therefore, aiming for the smallest possible nanoparticles might not be the best option (Bélteky et al., 2021). In the same vein, studies have shown that nuclear pore complexes have diameters ∼20–50 nm (Wente, 2000; Fahrenkrog & Aebi, 2003) and that nanoparticles with <50 nm size can enter the nucleus while those <35 nm can pass through the blood-brain barrier (Dawson et al., 2009; Hackenberg et al., 2011). Furthermore, maximum uptake efficiency has been reported for nanoparticles of sizes 30–50 nm (Huang et al., 2010; Varela et al., 2012; Gliga et al., 2014), and AgNPs of size 20–30 nm also showed no adverse effects and organ toxicity in animal studies (Lara et al., 2010; Recordati et al., 2015). Lastly, our previous studies showed that AgNPs with size 20–35 nm successfully delivered antiretroviral drugs to the brain and could be used as an appropriate nanodelivery method that can alleviate the effect of prolonged use of antiretroviral medications (Lawal et al., 2022a; Lawal et al., 2022b). Thus, one can suggest that optimal uptake efficiency or delivery for AgNPs could be achieved at sizes between 20 and 50 nm.

Apart from size, concentration is another factor affecting the activities of AgNPs. The work by Lau et al. showed that bacterial viability decreased as the concentration of AgNPs increased (Lau et al., 2017). Also, the Graphene oxide (GO) silver nanoparticles composite (GO–Ag) concentrations of 0.1, 0.5, 1.0 and 1.5 μg/ml showed no antibacterial activity against *Pseudomonas aeruginosa (P. aeruginosa)* bacteria. Thus, AgNPs concentrations of 2.5 and 5.0 μg/ml, which caused a significant decrease in cellular viability, were considered optimal biocidal concentrations for *P. aeruginosa* (de Faria et al., 2014). In producing AgNPs, the concentration should be kept low because the low concentration can achieve a therapeutic effect with little to no toxicity to human and animal cells. This is because even at low concentrations (0.0035 μg/ml 20 nm AgNPs and 0.22 μg/ml 80 nm AgNPs), AgNPs were taken up by retinal cells and disrupted retinal structure (Söderstjerna et al., 2014). Furthermore, soil enzymes were inhibited at higher AgNPs concentrations (Mishra et al., 2021), while low concentrations (0.06–0.12 mM) enhanced Horseradish Peroxidase activity (Karim et al., 2012).

Apart from size, the shape of the nanoparticles also affects their delivery and biological activity. The spherical shape is mostly favoured (Ghiuță and Cristea, 2020; Lawal et al., 2022b).

## 2 The brain as an human immunodeficiency virus reservoir site

Dating back to the 1980s, HIV has continued to exist as a global pandemic for nearly 40 years (Ash et al., 2021), affecting nearly 38 million people worldwide (Osborne et al., 2020). HIV is an infection that targets the body’s immune system, specifically the CD4 white blood cells, destroying these cells and weakening a person’s immunity against opportunistic infections (Vidya Vijayan et al., 2017). Significant progress has been made in the treatment of individuals living with HIV. However, the cure remains elusive to scientists and clinicians due to the yet-to-be-defined latent HIV reservoirs (stable accumulations of replication-competent forms of the virus in a myriad of anatomical sites throughout the body) (Ash et al., 2021; Blankson et al., 2002).

Some precise anatomical locations have been identified to harbour HIV infection. Those sites include the gut (oesophagus, duodenum, colon, etc.) and central nervous system (CNS), especially the brain (Chun et al., 2008; Wong and Yukl, 2016). The compartmentalisation of HIV within the brain allows for virus evolution with the possibility of evading clearance mechanisms and exhibiting a unique phenotype that only permits viral replication contingent upon immunosuppression (Bednar et al., 2015). The brain is not immune to HIV infection. Studies have identified that HIV invades the brain within 1–2 weeks of infection (Roberts et al., 2004; Valcour et al., 2012). In addition, the brain can subsequently serve as a sanctuary for ongoing HIV replication, even after systemic viral suppression has been achieved (Saylor et al., 2016). The perivascular macrophages and microglial cells are the productively infected cell types by HIV *in vivo* (Cosenza et al., 2002; Atluri et al., 2015). The neuro-invasion of HIV occurs both dependently and independently of a breakdown in the BBB through the frequent compromise of the BBB and infecting immune cells that can traffic into the brain by crossing the BBB, respectively (Osborne et al., 2020; Ash et al., 2021).

The BBB, as the name implies, is the barrier between the brain capillary blood and interstitial fluid of the brain (cells and other components that make up the brain tissue), ensuring a homeostatic environment (Banks, 2009; Dotiwala et al., 2020). The BBB came into existence in the late 19th century when Paul Ehrlich, a German Physician, noticed that a peripherally infused dye infiltrated all tissues except the brain and spinal cord. Thus, the BBB plays a vital role in controlling the influx and efflux of biological substances essential for the brain’s metabolic activity and neuronal function (Kadry et al., 2020). The BBB comprises capillary endothelial cells, basement membrane, neurological membrane, and glial podocyte (Dotiwala et al., 2020). The development of this barrier begins with angiogenesis, whereby pre-existing vessels, guided by vascular endothelial growth factor (VEGF), invade a developing neuro-ectoderm and give rise to new vessels that exhibit many BBB properties (Dotiwala et al., 2018). Unlike other endothelial cells, endothelial cells in the brain cavity present distinctive morphological, structural, and functional characteristics. These characteristics include the expression of tight junctions that seals the paracellular pathways between adjacent endothelial cells, permitting only a few selected types of substances between the cells. Other characteristics are the absence of fenestrations and lack of pinocytic activity, and expression of active transport mechanism (Ballabh et al., 2004; Dotiwala et al., 2018).

HIV habitually compromises the integrity of the BBB and infects the central nervous system in the early stages of infection (Osborne et al., 2020). In a study that used an *in vitro* BBB model, HIV infection of leukocytes caused an increase in the transmigration of HIV-infected cells across the BBB in response to the chemokine ligand 2 due to increased permeability, reduced tight junction proteins such as zonula occludens-1, claudin-1, and occludin in the BBB cells, and upregulation of matrix metalloproteinases-2 and -9 (Eugenin et al., 2006). Magnetic resonance imaging scans have revealed brain tissue alterations such as the expansion of the brain stem and third ventricle, loss of white matter integrity, and mild neurocognitive deficit within the first several months of CNS HIV infection (∼100 days) (Ragin et al., 2015). The persistent CNS-HIV infection and inflammation probably contribute to the development spectrum of neurologic or neurodegenerative diseases commonly referred to as HIV-associated neurocognitive disorders (HAND), which may affect more than 30% of people living with HIV regardless of virological suppression (Clifford and Ances, 2013). HAND can be categorised into three, each associated with an increasing level of impairment: 1) Asymptomatic neurocognitive impairment (30% prevalence)- shows HIV-associated impairment in cognitive function, but everyday functioning is not affected; 2) Mild neurocognitive disorder (20%–30% prevalence)- characterised by HIV-associated impairment in a cognitive function where interference in everyday functioning is displayed; and 3) HIV-associated dementia (2%–8% prevalence)- shows marked impairment in cognitive function, especially in learning of new information, information processing, and attention or concentration (Robertson et al., 2010; Eggers et al., 2017). Nonetheless, the incidence of HAND remains in the range of 20%–50% for people living with HIV (Eggers et al., 2017; Ash et al., 2021).

### 2.1 Neuroanatomy of the blood-brain barrier, human immunodeficiency virus and neurological disorders

The blood-brain barrier (BBB) plays a vital role in protecting the brain from pathogens, toxins and other harmful blood components, thereby maintaining the homeostasis between the blood and central nervous system (Le Guennec et al., 2019). The presence of biological barriers such as the blood-brain barrier and blood-cerebrospinal fluid barrier in the central nervous system has been proven to allow the penetration of HIV and other viruses but impede the adequate penetration of certain antiretroviral drugs (Rahimy et al., 2017; Warren, 2018). The BBB is formed by the astrocyte, pericyte, and endothelial cells linked together by tight junction proteins, which restrict the entry of specific molecules into the brain cells (Ortiz et al., 2014). The tight junction is regulated by signalling factors from blood microvascular endothelial cells as well as astrocyte and pericytes (Keaney and Campbell, 2015). The essential nutrients, such as amino acids, nucleotides, and carbohydrates, cross the BBB into CNS through the transport system, such as carriers and receptors (Ghalamfarsa et al., 2016). Also, more giant molecules cross the BBB through active transport systems such as P-glycoprotein, multi-resistance-associated proteins, L-transporters, and concentrative nucleoside transporter (Donaghue et al., 2014). Alteration in the BBB and increases in its permeability have been strongly linked with pathological acute and chronic CNS diseases (Yang et al., 2015). Likewise, HIV penetration and its replication in the CNS have been associated with various neurological disorders, such as behavioural, motor, and cognitive impairments (Qosa et al., 2015). HIV infiltrates CNS by viral transcytosis across the BBB endothelium and infects the target cells. This infection leads to neuroinflammation and brain damage, causing neurological disorders, as illustrated in Figure 2 (Lorin et al., 2020).

## 3 Antiretroviral drugs

In the past, HIV was considered a terminal illness, not until the development of Antiretroviral drugs (ARVDs). The availability of ARVDs to HIV patients has allowed them to live normally (Vance, 2010; Costagliola and AIDS, 2014). Over 40 ARVDs have been approved for use as of 2017 by the United States Food and Drug Administration (FDA). While the developing of new compounds has been slow for over a decade, many traditional compounds have made it to phase III clinical trials (Zhang, 2018). In the early years of HIV drug development, the available compounds could reduce viral load and increase CD4^+^ count, but a major challenge included drug toxicity (Vaidya et al., 2016; Zhang, 2018).

### 3.1 Antiretroviral drugs associated with neurological disorders

Treating HIV with ARVDs requires lifelong drug administration, predisposing the patients to develop certain pathologies, including neurological diseases, as listed in Table 1 (Bhatia and Chow, 2016). HIV-AIDS leads to progressive neurodegeneration of the immune and nervous systems with significant economic and health burdens (Ahmadu et al., 2022). HIV targets the central nervous system (CNS), where it replicates and may persist for a long among untreated seropositive patients (Varatharajan and Thomas, 2009). The prevalence of neurocognitive impairment associated with HIV varies between 25% and 70%, depending on the profile of the study population and the neurocognitive impairment (NCI) definition (Heaton et al., 2011; Wright et al., 2015; Santos et al., 2019).

**TABLE 1:** Summary of neurological effects of ARVDs.

	Class	Name	Neurological effect(s)	Specie(s)	References
1	NNRTIs	Efavirenz	Encephalopathy and cerebellar ataxia	Humans	Sarma et al. (2022)
2	NNRTIs	Nevirapine	Reversed working memory deficits; increased exploratory activities; reduced cholinesterase and glutamate activities; increased antioxidant profile in middle cerebral artery occlusion (MCAO) model of ischemic damage	Wistar rats	Pathakala et al. (2016)
3	NNRTIs	Nevirapine	Increased motor activity; pilo-erection; Straub-tail phenomenon; grooming	NMRI mice	Ahmadu et al. (2022)
4	NNRTIs	Nevirapine	Delayed sleep onset and decreased sleep duration in diazepam-induced sleep; Prolonged ketamine-induced sleeping time	NMRI mice	Ahmadu et al. (2022)
5	NRTIs	Tenofovir disoproxil fumarate (TDF)	Worsened working memory in diabetic rats	Sprague-Dawley rats	[S. Lawal et al. (2022a)]
6	NRTIs	Tenofovir disoproxil fumarate (TDF)	Increased lipid peroxidation; neuroinflammation, reduced astrocytic GFAP-positivity; increased mitochondrial damage in the prefrontal cortex of diabetic rats	Sprague-Dawley rats	[S. Lawal et al. (2022b)]
7	cART	Emtricitabine + Tenofovir disoproxil fumarate	Induced oxidative damage; promoted autophagy; reduced hippocampal neuroplasticity	BALB/c mice	Zulu et al. (2018)
8	NNRTIs	Nevirapine	Increased hippocampal astrocyte GFAP positivity; neuroinflammation; reduced BDNF expression	BALB/c mice	Zulu et al. (2018)
9	NRTIs	Tenofovir disoproxil fumarate (TDF)	Increased hippocampal astrocyte GFAP positivity; increased neuroinflammation	BALB/c mice	Zulu et al. (2018)

MDA, malondialdehyde; IL-1β, interleukin-1, beta; GPx, glutathione peroxidase.

ARVDs, which have a high capacity to penetrate the CNS, would serve as a promising treatment strategy to control HIV replication while reducing the risk of developing NCI (Santos et al., 2019). CNS penetration-effectiveness score (CPE Score) is used to assess the capacity of ARVDs to penetrate the CNS, inhibiting viral replication. Therefore, a high CPE score favours a reduction in CSF HIV RNA (Letendre et al., 2008; Cusini et al., 2013). Although a high CPE has been associated with reduced CNS HIV RNA replication (Letendre et al., 2008; Cusini et al., 2013), some authors have found a negative association or no association between CPE scores and NCI (Marra et al., 2009; Simioni et al., 2010; Caniglia et al., 2014; Baker et al., 2015). A negative association between CPE scores and NCI has been attributed to the neurotoxic effect of some ARVDs (Robertson et al., 2012). Despite the systemic effects of ARVDs, there is an increased presence of HIV in the CNS. The increased presence might be due to the inefficiency of ARVDs in crossing the BBB (Cunningham et al., 2000; Ene et al., 2011). Besides, the efficiency of ARVDs is only determined by plasma HIV RNA level (Varatharajan and Thomas, 2009; Ene et al., 2011).

The development and introduction of HAART have led to a significant reduction in neurological comorbidity associated with AIDS (Ganau et al., 2012; Sacktor et al., 2016). Although some of these diseases can result from HIV infection associated with prolonged inflammation, there is evidence that ARVDs toxicity can also contribute to the development of these comorbidities (Bertrand et al., 2016). Several side effects have been linked to ARVDs use, including peripheral neuropathy and mental disorders (Chen et al., 2013; Margolis et al., 2014). For instance, there is evidence of inflammation and neurotoxicity following non-nucleoside reverse transcriptase inhibitors (NNRTIs) use (Diaz-Delfin et al., 2011).

Also, HAART has been linked with mitochondrial damage and subsequently increases the risk of neuropathy and neuroinflammation (Lin et al., 2018). Chronic administration of HAART to HIV-positive patients has been reported to cause neuroinflammation, changes in astrocyte mitochondrial membrane and mitochondrial ROS production (Cohen et al., 2017). The continuous use of HAART to prevent a viral rebound in people living with HIV and diabetes causes neuroinflammation with detrimental effects on CNS astrocytes. This contributes significantly to neuropathologies aetiology (Yang et al., 2015; Cohen et al., 2017; Yirong). Excessive production of pro-inflammatory cytokines during neuroinflammation has been implicated in cognitive deficits and anxiety disorders (Charlton and Starkey, 2018; Li et al., 2019). Interestingly, in the post-HAART era, people living with HIV have experienced an improvement in motor skills and verbal fluency, but executive functions and anxiety-like behaviour remain dominant impairments (Heaton et al., 2011; Checa et al., 2020). In addition, the prevalence of anxiety and depression among the patient receiving HAART remains high (Rabkin et al., 2000). The most used components of HAART (Efavirenz and Tenofovir) have been reported to cross the blood-brain barrier, causing mitochondrial dysfunction and some neurological-related adverse effects like depression and anxiety (Chen et al., 2019; Checa et al., 2020). In contrast, another study that investigated the effect of HAART (Efavirenz and Ritonavir) on HIV-negative patients found no significant effect of the drugs on functional connectivity or cerebral blood flow. The authors concluded that functional connectivity and cerebral blood flow in HIV-positive patients receiving HAART are not due to the drugs but to the virus (Brier et al., 2015). In addition, HIV patients also have a higher incidence of cerebrovascular diseases when compared to non-infected patients (Ovbiagele and Nath, 2011; Benjamin et al., 2012). HIV-infected patients with stroke often exhibit different characteristics from others with lesser symptoms, e.g., high blood pressure (Chow et al., 2012). In Wistar rats, Bakshi and colleagues showed that Nevirapine had a neuroprotective effect against ischemic stroke in middle cerebral artery occlusion (MCAO) (Pathakala et al., 2016).

Furthermore, a recent study found that the administration of Nevirapine increased the number of line crossings in the open field test (OFT), resulting in locomotor activities in experimental mice (Ahmadu et al., 2022). An increase in locomotor activity in the context of the OFT suggests anxiolytic effects (Iorjiim et al., 2020). Similarly, a previous study found that Nevirapine administration reversed MCAO-induced reduction in open-field exploration (Pathakala et al., 2016). In another study where cART (Emtricitabine + Tenofovir disoproxil fumarate) was administered for 8 weeks, no changes in exploratory activity were observed (Zulu et al., 2021). A recent preclinical study found that administering Nevirapine increased other gross behaviour, including piloerection, the Straub-tail phenomenon and grooming (Ahmadu et al., 2022). There is evidence of delayed Efavirenz neurotoxicity (Sarma et al., 2022). Sarma and colleagues recently studied an HIV patient placed on HAART (Tenofovir 300 mg, Lamivudine 150 mg and Efavirenz 600 mg) for about 3 years. When they applied Naranjo’s algorithm, they found a score of 6, indicating Efavirenz’s toxicity (Naranjo et al., 1981; Sarma et al., 2022). They later considered Efavirenz-induced encephalopathy and cerebellar ataxia with HIV-related polyneuropathy as possible diagnosis (Sarma et al., 2022). The underlying mechanism of action of the neurotoxic effect of Efavirenz is unclear. However, oxidative stress has been suggested to contribute to its adverse effects (Lanman et al., 2021). Also, sleep dysfunction has been associated with ARVDs in rodents. A recent study by Ahmadu and colleagues showed that the administration of Nevirapine delayed sleep onset and decreased sleep duration in diazepam-induced sleep in mice (Ahmadu et al., 2022).

Moreover, there is evidence of cognitive deficits following ARVD administration. A previous study by Bakshi and colleagues found that Nevirapine administration reversed middle cerebral artery occlusion (MCAO)-induced working memory deficits in rats (Pathakala et al., 2016). In another study where cART (Emtricitabine + Tenofovir disoproxil fumarate) was administered for 8 weeks, it was observed that cART increased escape latencies in the Morris water maze test for spatial memory, indicating cognitive impairment (Zulu et al., 2021). A recent study by Lawal and colleagues found no effect of HAART (a fixed-dose combination of Efavirenz, emtricitabine, and tenofovir disoproxil fumarate) administration on spatial and working memory in rats (Lawal et al., 2021). In another study by Lawal and colleagues, they also found that the administration of Tenofovir disoproxil fumarate (TDF) alone did not alter working memory in rats as tested in Y-maze. However, TDF administration worsened the working memory of diabetic rats (S. Lawal et al., 2022b).

In addition, several neurochemical changes have been observed following ARVD administration. In a preclinical study of ischemic stroke, Nevirapine administration offered neuroprotective effects by reducing hippocampal levels of acetylcholinesterase (AChE) and glutamate following MCAO in rats (Pathakala et al., 2016). Zulu et al. (2018) administered either Nevirapine or Tenofovir disoproxil fumarate for 8 weeks and observed that Nevirapine or Tenofovir disoproxil fumarate administered separately increased the number of GFAP-positive cells, IL-1β concentration and TNF-α expression in the hippocampi of mice. However, Nevirapine but not Tenofovir disoproxil fumarate reduced BDNF expression (Zulu et al., 2018). In another study by Zulu et al. (2021), it was also found that prolonged cART treatment induced oxidative damage by increasing 4-hydroxynonenal concentration in the hippocampus of mice. Also, treatment with cART promoted autophagy *via* increased expression levels of LC3B-II and reduced hippocampal neuroplasticity *via* downregulation of BDNF and synaptophysin (Zulu et al., 2021). Nevirapine administration also offered antioxidant effects by increasing brain levels of catalase and GPx following MCAO in rats (Pathakala et al., 2016). In contrast, it was observed that while TDF administration did not alter the Malondialdehyde (MDA, a marker of lipid peroxidation) and interleukin-1 beta (IL-1β, a pro-inflammatory cytokine) in the prefrontal cortex of normal rats, TDF increased MDA and IL-1β levels in the prefrontal cortex of diabetic rats when compared to rats that did not receive TDF (S. Lawal et al., 2022a). In the same study, an ultrastructural assessment of the prefrontal cortex revealed that diabetic rats that received TDF had reduced GFAP-positive astrocytes and increased mitochondrial damage (S. Lawal et al., 2022b).

### 3.2 Antiretroviral drugs and blood-brain barrier

The blood-brain barrier is an effective mechanism of the brain to regulate the influx and efflux of substances, hence protecting the brain from toxins and pathogens reaching the periphery (Qosa et al., 2015). The BBB consists of endothelial cells, astrocytes and pericytes, which work together to interact with the neurons. The endothelial cells are composed of tight junctions that control the passage of molecules in and out of the neurons (Daneman and Prat, 2015; Keaney and Campbell, 2015). Several acute and chronic diseases can impair the BBB, allowing immune cells into the CNS, inducing inflammation, neuronal apoptosis, and the influx of other pathogens. Dysregulated influx of molecules in the CNS can lead to the development of Alzheimer’s Disease or Stroke (Obermeier et al., 2013).

Of all the diseases associated with HIV, cerebrovascular diseases are the leading cause of death (Aldaz et al., 2011; Cima et al., 2016). A study that examined the role of NNRTIs on BBB integrity found that out of all the NNRTIs tested, only Efavirenz altered endothelial integrity by downregulating claudin-5 expression. Furthermore, they showed that this impaired BBB exacerbated tissue damage in stroke (Bertrand et al., 2016). Thus, evidence suggests that the choice of ARVDs is essential to avoid neurotoxicity, eventually leading to a decline in treatment outcomes (Bertrand et al., 2016).

Despite a relationship between CPE scores and NCI, a high CPE does not necessarily correlate with reduced neurocognitive impairment (NCI) (Peluso et al., 2012). While some authors found that a high CPE score was associated with reduced NCI (Ciccarelli et al., 2013; Vassallo et al., 2014; Carvalhal et al., 2016), others found no association, including a negative association (Marra et al., 2009; Simioni et al., 2010; Caniglia et al., 2014; Baker et al., 2015) between CPE scores and NCI.

Nevirapine belongs to the class of non-nucleoside reverse transcriptase inhibitors (NNRTIs) of ARVDs and has the highest CPE score (Antinori et al., 2005; Nwogu et al., 2016). Nevirapine acts by inhibiting the reverse transcriptase enzyme responsible for the reverse transcription of HIV RNA to DNA (Ahmadu et al., 2022).

## 4 Recent advances and prospects

### 4.1 Nanoparticles in drug delivery

**FIGURE 5:**
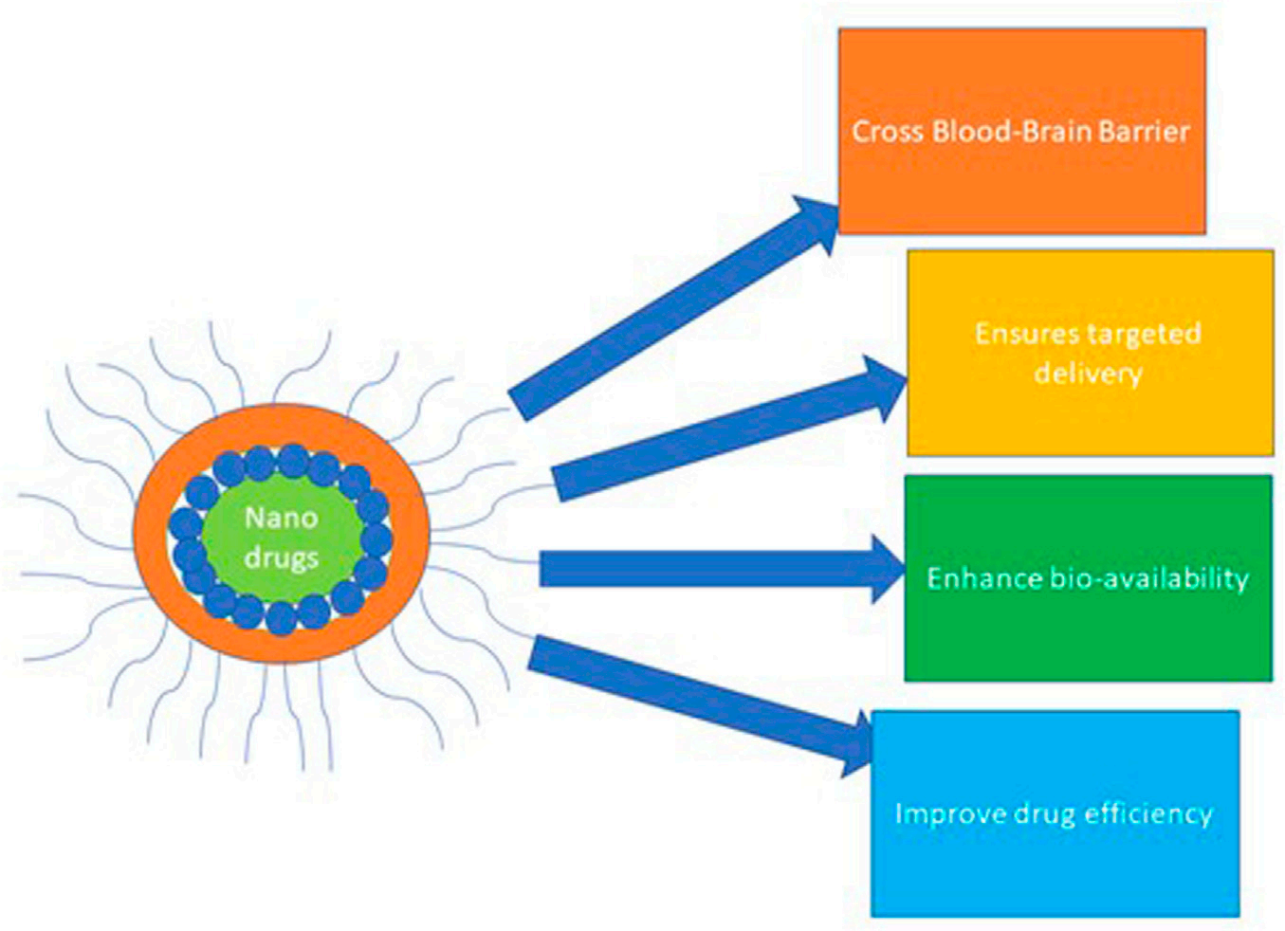
Nanoparticle conjugated drugs in the drug delivery system.

The application of nanoparticles (NPs) to diagnose, treat and control various diseases represents a tremendous advancement in nanotechnology and nanomedicine (Mohamed et al., 2022). Notably, a nanoparticle drug delivery system (NDDS) is a remarkable advancement in nanomedicine that employs nano-size particles to diagnose and control the delivery of various curative agents to the target cell/tissue (Patra et al., 2018). The nanoparticle delivery system ensures targeted and sustained delivery, enhanced drug bioavailability, and improved drug pharmaco-efficiency, as illustrated in Figure 5. Thus, its road application in delivering immunotherapeutic, chemotherapeutic, and biological agents in managing diseases (Ventola, 2017; Patra et al., 2018; Naidu et al., 2021; Mohamed et al., 2022).

### 4.2 Choice of nanoparticles receiving attention in the delivery antiretroviral drugs to the brain

**FIGURE 6:**
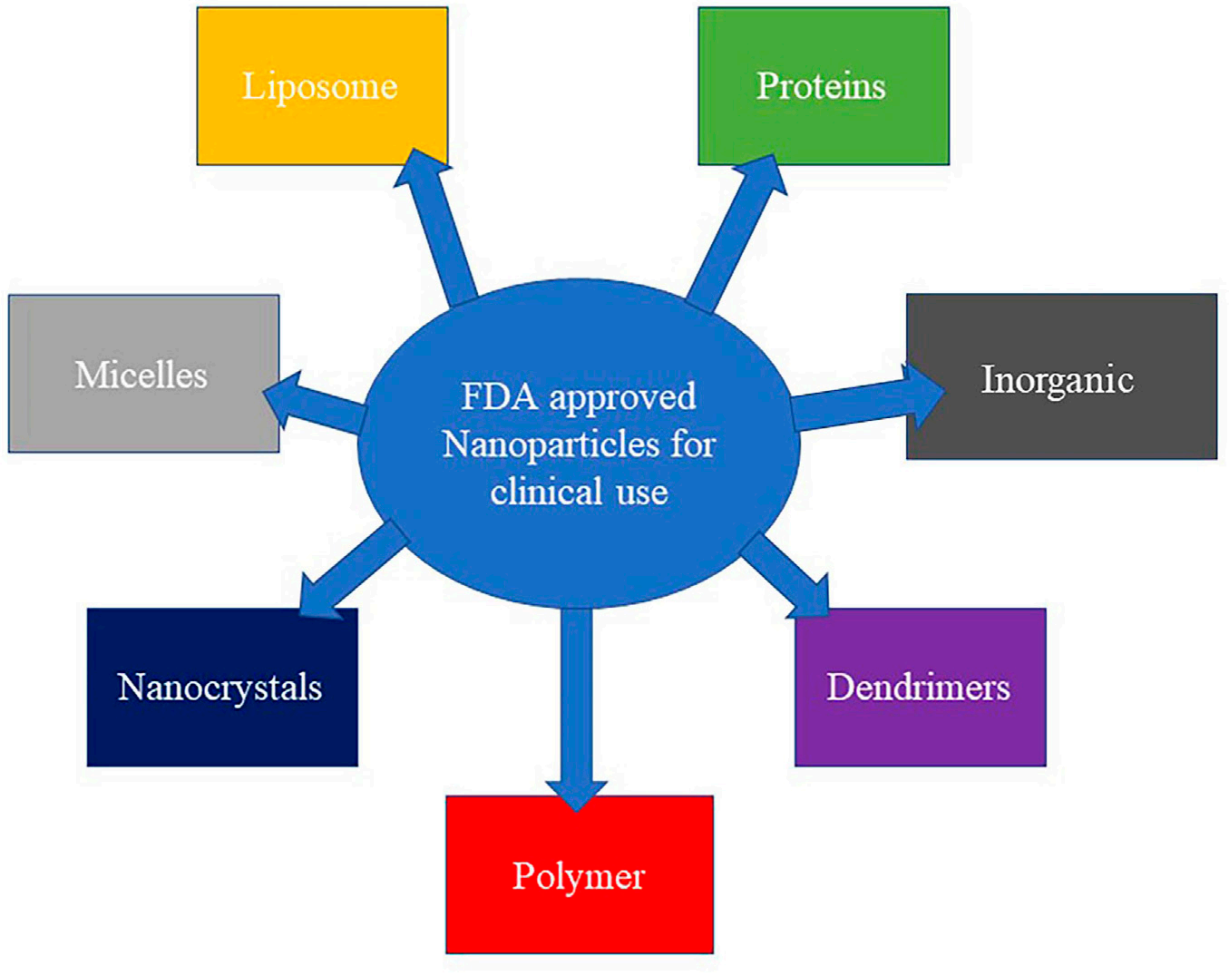
US FDA’s approved nanoparticles for clinical use.

Growing research evidence has revealed the blood-brain barrier (BBB) as a significant obstacle in managing neurological disorders because it hinders major neurological therapeutic agents (Zhu et al., 2019). As a result of this, various classes of the US Food and Drug Administration (FDA) approved NPs have been investigated in animal studies and clinical trials for their ability to cross the BBB and ensure therapeutic efficiency, as shown in Figure 6 (Bobo et al., 2016; Wen et al., 2017; Van Norman, 2019). Of NP classes, some polymeric- and lipid-based NPs have received approval from FDA, some are undergoing approval processes, and others are available for clinical use (Sainz et al., 2015; Bobo et al., 2016; Ventola, 2017). In clinical investigations, examples of the approved liposome-based nano-drug are liposomal vincristine for general use, protectant alfa for respiratory distress syndrome, liposomal cytarabine for lymphomatous meningitis, and doxorubicin HCl liposome injection for managing Karposi’s sarcoma (Bobo et al., 2016; Caster et al., 2017; Ventola, 2017). Other liposome-based nanodrugs are liposomal amphoteric B and B complex for managing fungal infections and liposomal morphine sulphate for managing postoperative analgesia. In addition, liposomal Irinotecan, liposomal verteporfin and liposomal daunorubicin were approved for managing pancreatic cancer, ocular histoplasmosis, and acute myelogenous leukaemia (Ventola, 2017).

Previously, Chen et al. (2016) formulated transferrin-based liposomes to deliver α-Mangostin across the blood-brain barrier and improve the existence of cerebral cortical neurons in rats. This formulated α-Mangostin was delineated as a possible therapeutic agent in managing Alzheimer’s disease (Chen et al., 2016). More so, a liposome-modified agent was intravenously delivered and crossed BBB to selectively bind Amyloid-β plaque deposits in APP/PSEN1 Transgenic Mice (Tanifum et al., 2012).

Polymer micelles represent other FDA-approved NPs for drug delivery purposes. In recent times, synthetic polymers such as poly (N-isopropyl, bio-absorbable polymers, dendritic polymers, acrylamide), poly (2-hydroxyethyl methacrylate), biodegradable, and poly (ethylenimine) have received great attention in drug delivery (Sung and Kim, 2020). Likewise, several natural polymers that include dextrin, polysaccharides, hyaluronic acid, chitosan, poly (lactic acid), arginine, and poly (glycolic acid) have been employed to load and deliver therapeutic agents (Sung and Kim, 2020; Damiri et al., 2022). Polymeric NPs are characterised by targeted delivery, sustained release, and enhanced drug effectiveness (Patel et al., 2013). More so, advancement in formulating vehicle-enhanced transportation of nanoparticle-loaded drugs across the biological barrier like the BBB is gaining wide priority and has provided a clue on the targeted delivery to various parts of the brain (Cacciatore et al., 2016; Teleanu et al., 2018).

Dendrimer nanoparticles represent other NPs currently attracting attention in drug delivery systems due to their high solubility, multivalency, chemical stability, electrostatic interactivity, uniform size, and low cytotoxicity (Abbasi et al., 2014). Recently, a poly (amido) amine dendrimer conjugated streptavidin and poly-amidoamine dendrimers were formulated and investigated on murine and mouse models. These conjugates demonstrated an excellent permeation into the central nervous system to deliver the therapeutic agent in managing neurological disorders (Srinageshwar et al., 2017; Moscariello et al., 2018). Moreso, remarkable results have been obtained in managing Parkinson’s and Alzheimer’s diseases by using dendrimer tesaglitazar composite to induce a phenotype interaction of glia cells and improved β-amyloid phagocytosis (DeRidder et al., 2021).

Interestingly, inorganic/metallic nanoparticles have been widely investigated for their unique properties, which gives them an edge to be used in delivering therapeutic agents across BBB and ensuring effective management of neurological disorders. Tisch et al. (2013) reported fast, cost-effective and dependable biomarkers for Parkinson’s disease (PD) and Alzheimer’s disease (AD) using functionalised carbon nanotubes and gold nanoparticle-based sensors. In a similar study, Sonawane and colleagues fabricated protein-capped metal NPs (PC-CdS and PC-Fe_2_O_4_) and explored their potential therapeutics against protein accumulation in AD (Sonawane et al., 2019). Their findings, especially with PC-CdS, revealed excellent inhibition of Tau aggregation and suggested a potent AD therapeutic delivery.

The limitations and the circumstances surrounding the conventional therapeutics of neurological disorders such as epilepsy, AD, PD and Huntington’s disease have directed the research attention and biomedical application to the usage of metallic NPs to detect and manage these disorders (Haque and Patra, 2022). Of these classes of NPs, gold NPs bioconjugates have been extensively used to deliver drugs to the brain in attempts to manage neurological disorders (Mirsadeghi et al., 2015; Soursou et al., 2015; Sivanesan and Rajeshkumar, 2019; Talebpour and Ghahghaei, 2020; Xu et al., 2021). Also, Parveen et al. (2022) reported an exceptional therapeutic efficacy of biosynthesised silver NPs (AgNPs) against PD and AD. However, despite their unique characteristics, there is a paucity of attention and application to AgNPs in neurological therapeutics delivery.

### 4.3 Silver nanoparticles as a promising candidate for delivering antiretroviral drugs to the brain

The upsurge in neurological disorders is becoming a public health issue of grave concern and requires biomedical researchers’ attention (WHO, 2007; Naqvi et al., 2020). Previously, many therapeutic modalities have been investigated and employed, but the effectiveness of these treatment regimens could not provide a solution to these neurodegenerative disorders (Ghosh and De, 2020). The central nervous system-associated biological barriers that limit the efficacy of these therapeutic modalities include; the blood-cerebrospinal fluid barrier and BBB (Naqvi et al., 2020). These barriers regulate the influx and efflux of drugs and substances in and out of the brain (De Boer et al., 2003; Löscher and Potschka, 2005). For this reason, the selectively permeable nature of the BBB has been described as a significant setback in the treatment of neurodegenerative disorders (Lawal et al., 2018).

As the research into the effective therapeutic modality for neurodegenerative disorders intensifies, the nanoparticle drug delivery system (NDDS) is now receiving broad research attention. Growing research evidence has shown that nanoparticle-loaded drugs have higher drug bioavailability, better penetration of biological barriers, better distribution, and higher metabolism (Kaur et al., 2016). AgNPs serve as a candidate to deliver therapeutic agents through BBB to reach the brain effectively. AgNPs can effectively deliver therapeutic agents through BBB to the brain based on their size, sustained drug delivery, and physicochemical antioxidant, anti-inflammatory and antimicrobial properties (Al-Obaidi et al., 2018; Jiang et al., 2018; Karthik et al., 2018; Selvan et al., 2018).

Previously, the effects of biosynthesised AgNPs against amyloid build-ups were explored, and significant inhibition of amyloid fibril build-ups was reported, suggesting an effective treatment modality in managing amyloidosis diseases (Dehvari and Ghahghaei, 2018). The mechanistic understanding of the pathogenesis of most neurological disorders was linked to the upregulation of the amyloid-beta (Aβ) aggregation. Aβ represents peptide amino acids, the derivatives of the amyloid precursor proteins (APP), and their upregulation causes neurological disorders like AD (Dong et al., 2012). We hypothesise that since AgNPs have been shown to inhibit Aβ aggregation, they could be useful in delivering therapeutic agents across BBB and cater for neurological disorders.

### 4.4 Toxicity profile of silver nanoparticles: Minimizing the toxicity to exploit their features in neurological therapeutics

Despite many attempts to use metallic NPs to deliver drugs to the CNS with outstanding results, the safety of these metallic NPs, especially AgNPs, has raised concerns as experts advise about their potential toxicities. The cause of the AgNPs’ toxicity could be the ease at which they undergo oxidation by oxygen and other substances in the body system or environment, leading to the release of silver ion (Ag+), a well-known toxic ion. Ag + has been described as a significant cause of AgNPs’ toxicity (Bar-Ilan et al., 2009; McShan et al., 2014). Also, previous studies have revealed that the toxicity of AgNPs is size-dependent. For example, more cytotoxicity was observed at the smaller size (2–4 nm) of AgNPs in macrophage cells compared to medium size (5–7 nm) and larger size (20–40 nm) (Yen et al., 2009). Also, embryonic zebrafish exposed to smaller-sized (3 nm) AgNPs displayed higher malformation and mortality than those that were exposed to a larger size (10–50 nm) (Bar-Ilan et al., 2009). In addition, a study revealed that smaller size (5 nm) AgNPs were cytotoxic to 4 cell lines, SGC-7901, HepG2, A549 and MCF-7, than 20–50 nm AgNPs (Liu et al., 2010).

Another factor contributing to the AgNPs’ toxicity is their shape. Based on existing evidence, spherical shape AgNPs are mainly non-cytotoxic. A study that compared wire AgNP and spherical AgNPs in A549 cells showed no deleterious effect of spherical AgNP but cytotoxicity of wire AgNP (Mahmuda Akter et al., 2018; Stoehr et al., 2011). The route of administration of the synthesised AgNPs represents another factor responsible for various toxicity profiles of AgNPs. There are diverse routes of NPs’ entry into the body system. NPs can be inhaled, ingested, transdermally applied, intraperitoneally or intravenously injected (De Matteis, 2017). However, studies have shown that intraperitoneal, intravenous and subcutaneous administration of AgNPs allows AgNPs to gain direct access to systemic circulation and has thus been the most adopted route in AgNP-based therapeutic vehicles (Ferdous and Nemmar, 2020). Notably, the characterisation of AgNPs is an essential step that involves the evaluation of the size, shape, forms, charge, ion release, level of aggregation, functional groups, particle dissolution, and the elemental composition of the synthesised AgNPs. Several studies have ascribed the proper biological activity of AgNPs to the characterisation (Froggett et al., 2014; Wei et al., 2015; Zhang H., 2016; Kim et al., 2017).

**FIGURE 7:**
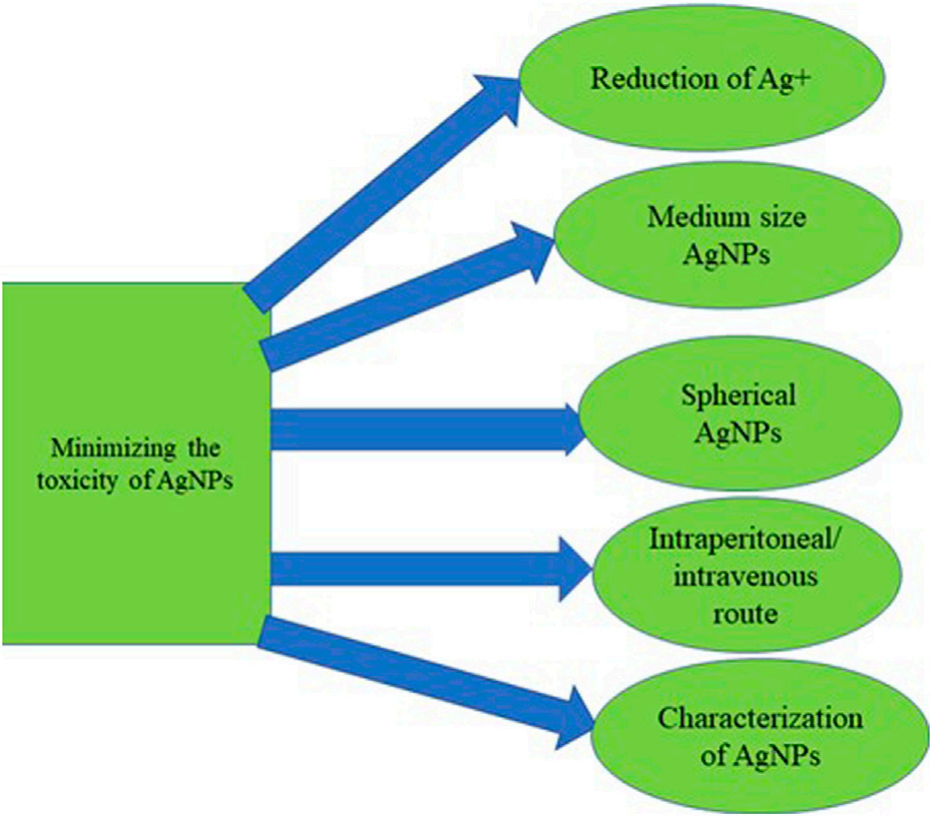
Factors required for minimising AgNPs toxicity while exploiting their unique features.

The mechanism for the AgNPs toxicity has alluded to the ability of Ag + to generate free radicals in the brain, which serves as the primary target of metallic NPs upon entering the systemic circulation with potential neurotoxicity through apoptosis, inflammation, and free radicals radical-induced oxidative stress (Song et al., 2016). The summary of the factors that could minimize the toxicity profile of silver nanoparticles is illustrated in Figure 7.

## 5 Conclusion and recommendations

Despite the development of effective ART, the neurocognitive impairment associated with HIV remains a challenge, and there is still a dearth of information regarding the neuropharmacological effects of ARVDs (Ahmadu et al., 2022). Furthermore, many side effects of ARVDs, such as sleep disturbance or prolonged wakefulness, reported as feedback from clinicians are mainly unpublished. Similarly, there is underreporting of patients’ experiences following ARVDs administration (Ahmadu et al., 2022), which challenges the development of targeted HIV/AIDS therapies. It is imperative to manage and reduce neurological complications of AIDS in HIV patients. Although there is an ongoing development of better therapeutic strategies to improve HIV treatment, therapies targeted at neurological disorders are insufficient. Therefore, nanoparticle-targeted drug delivery could reduce the neurological effects of ARVDs and improve their efficiency. AgNPs have been widely used in drug delivery, but their use has been subjected to scientific deliberations due to their perceived toxicity.

We hypothesise that the toxicity of AgNPs could be minimised by using appropriate reducing and stabilising agents such as trisodium citrate to reduce silver ion Ag + to ground state Ag^0^ during the synthesis. More so, usage of medium size, spherical-shaped AgNPs is encouraged in AgNPs-based drug delivery to the brain due to their ability to deliver therapeutic agents across BBB. In addition, characterisation and functionalisation of the synthesised AgNPs are required during the drug delivery approach. In addition, understanding the pharmacokinetics and cellular uptake of AgNPs as well as how the properties of AgNPs affect the pharmacokinetics of the therapeutic agents would be a major breakthrough in drug delivery. Putting all these factors in place would minimise toxicity and enhance the usage of AgNPs in delivering therapeutic agents across the BBB to the targeted brain tissue and could cater for the HIV-associated neurocognitive disorders and neurotoxic effects of ARVDs.
